# Placental transfer and tissue accumulation of dolutegravir in the *ex vivo* human cotyledon perfusion model

**DOI:** 10.1371/journal.pone.0220323

**Published:** 2019-08-13

**Authors:** Laurent Mandelbrot, Pierre-François Ceccaldi, Dominique Duro, Minh Lê, Lucile Pencolé, Gilles Peytavin

**Affiliations:** 1 Assistance Publique-Hôpitaux de Paris, Hôpital Louis Mourier, Service de Gynécologie-Obstétrique, Hôpitaux Universitaires Paris-Nord Val de Seine, Colombes, France; 2 Université Paris-Diderot, Université Sorbonne Paris-Cité, Paris, France; 3 Département Hospitalier Universitaire Risques et Grossesse, Paris, France; 4 IAME, UMR 1137, INSERM, Paris, France; 5 Assistance Publique-Hôpitaux de Paris, Hôpital Beaujon, Service de Gynécologie-Obstétrique, Hôpitaux Universitaires Paris-Nord Val de Seine, Clichy, France; 6 Agence Nationale de Recherches sur le Sida et Hépatites Virales (Inserm-ANRS), Paris, France; 7 Assistance Publique-Hôpitaux de Paris, Hôpital Bichat-Claude Bernard, Pharmaco-Toxicology Department, Hôpitaux Universitaires Paris-Nord Val de Seine, Paris, France; University of Mississippi Medical Center, UNITED STATES

## Abstract

**Objective:**

To determine the transplacental pharmacokinetics of the HIV integrase inhibitor dolutegravir.

**Study design:**

Maternal-to-fetal transfer across the term human placenta was investigated with the ex-vivo dually perfused cotyledon model, in 5 closed-circuit, recirculating experiments. Dolutegravir was added to a maternal perfusate containing antipyrine, a marker to validate the cotyledon’s viability, and 2 g/liter of human albumin.

**Results:**

After 3h of recirculating perfusion, the mean (± SD) DTG concentrations in the maternal and in the fetal compartments were respectively 2450 ± 286 ng/mL and 715 ± 369 ng/mL, with a fetal-to-maternal ratio of 34% ± 18% and a clearance index (in comparison with antipyrine transfer) of 79% ± 23%. The mean cotyledon accumulation index was 153% ± 25%.

**Conclusion:**

Fetal transplacental exposure to dolutegravir was considerable as well as accumulation in placental tissue. Whether this may lead to risks for the exposed fetus requires more investigation.

## Introduction

Sustained suppression of plasma viral load is the key to preventing vertical transmission from HIV-infected women to their children. In the prospective multicenter French Perinatal Cohort (ANRS-EPF), no mother-to-child-transmission of HIV-1 occurred among nearly 3000 women receiving antiretroviral therapy (ART) before conception and throughout the pregnancy, with a plasma VL <50 copies/mL at delivery [1]. Antiretroviral therapy is clearly recommended for all HIV infected pregnant women [2], but the choice of which medications to use must take into account the tolerance for the developing fetus, in view of the placental transfer of the drugs used [3].

Dolutegravir is an HIV-1 integrase strand transfer inhibitor (INSTI) which is increasingly used and recommended by the World Health Organization for a universal first-line of ART, in combination with nucleoside reverse transcriptase inhibitors or rilpivirine [4]. Whereas early first reports of use of dolutegravir in HIV-1-infected pregnant women were reassuring [5–10], a large prospective study in Botswana showed an increased risk of neural tube defects in infants born to women taking dolutegravir at the time of conception [11, 12].

Because clinical trials are ethically difficult in pregnant women, other preclinical approaches are required in order to obtain data on the exposure of the fetus to medications given to the mother. The ex vivo human cotyledon is an accepted model used to study and interpret placental transfer of drugs [13–18].

The purpose of this study was to investigate the placental transfer of dolutegravir in the ex vivo human perfused cotyledon.

## Materials and methods

Placentas were collected after uncomplicated pregnancy with full-term delivery (≥ 37 weeks gestational age) in mothers who were seronegative for HIV and hepatitis B and C viruses and had taken no medication other than vitamin supplements and perimedullar analgesia. They were collected in the maternity ward of Louis Mourier Hospital, Assistance Publique-Hôpitaux de Paris, Colombes, France, and were rapidly perfused on site. Women who donated a placenta gave their written informed consent. The research project was approved by the Institutional Review Board (Comité d’Ethique et d’Evaluation dans la Recherche Biomédicale, IRB N° IRB00006477, May 21, 2010).

Dolutegravir was obtained by crushing commercial tablets (each film-coated tablet of Tivicay contains 50 mg of dolutegravir sodium), after which the powder was weighed, solubilized in dimethyl sulfoxide (DMSO, Merck, Darmstadt, Germany) and prepared in separate tubes, in order to contain 3 mg of dolutegravir for each experiment. The target was to obtain, after dilution in the maternal compartment, concentrations for the active drug as observed in adults taking dolutegravir 50 mg once daily, where Cmax is on the order of 3000 ng/mL [19]. Experiments were conducted as described previously [20–23]. Perfusates were prepared with Earle’s Balanced Salt Solution, with the addition of human albumin at 2 g/liter (Ydrabulmin 20%, LFB-Biomedicament, Courtaboeuf, France). Then, dolutegravir was added into the infusion reservoir on the maternal side, as well as antipyrine at 20 mg/liter, as a marker to validate the cotyledon’s viability [24]. Antipyrine, phosphate-buffered saline and Bradford reagent were purchased from Sigma-Aldrich (Saint Quentin Fallavier, France), other reagents from Invitrogen (Cergy-Pontoise, France). The perfusion method was adapted from Schneider et al. [25], as previously described [23, 26, 27]. Perfusions were started within 20 min after delivery. An intact isolated cotyledon was selected, then a fetal chorionic plate artery and its associated vein were cannulated. A significant pressure in the fetal artery was required to consider that the cotyledon was viable and start the perfusion. The maternal side was then examined for whitening of the perfused area, and the selected cotyledon was placed in the perfusion chamber with the maternal side up. The intervillous space on the maternal side was perfused using two needles piercing the basal plate. Each circuit was pumped separately by a peristaltic pump (Minipuls 3; Gilson Medical Electronics, Villiers le Bel, France). The fetal and maternal flow rates were 6 and 12 ml/min, respectively, and arterial pressures were monitored throughout the experiments. The system reached steady state after 15 to 30 min. The pH values of the maternal and fetal solutions were adjusted to 7.40. and 7.20, respectively, and the temperature was maintained at 37°C.

Recirculating/closed perfusions were performed. The perfusate which leaked from the intervillous space into the perfusion chamber was recirculated into the maternal reservoir.

Samples were collected every 5 min over 180 minutes to determine the concentrations of dolutegravir and antipyrine in the fetal and maternal compartments and stored at -20°C until analysis. Dolutegravir concentrations in maternal and fetal samples were determined using ultraperformance liquid chromatography coupled with tandem mass spectrometry (Waters Xevo UPLC-TQD, Milford, MA, USA) [28]. Analyte separation was accomplished using a Waters Acquity UPLC BEH C18, 50 ×2.1 mm column, with a particle size of 1.7 μm. A gradient mobile phase method was used, composed of 0.05% formic acid in water (A) and methanol (B). The lower limit of quantification was 5 ng/mL for dolutegravir. Antipyrine concentrations were determined by high-performance liquid chromatography with UV detection at 290 nm after liquid-liquid extraction. The analytic column for antipyrine separation consisted of an octadecylsilyl NovaPak (3.9 mm by 150 mm). The mobile phase comprised 0.05 M phosphate buffer (pH 3)-methanol-tetrahydrofuran (75:25:0.9 [vol/vol/vol]). Standard curves for antipyrine concentrations ranged from 0.5 to 20 mg/L. The lower limit of quantification for antipyrine was 0.01 mg/L. We calculated according to the formulas of Challier et al [24] the fetal transfer rate (FTR), defined as the ratio of fetal to maternal concentrations at steady state, and the clearance index (CLI), defined as the ratio of the FTR of the study drug over the FTR of antipyrine [24]. An antipyrine FTR of >20% was required to validate each experiment. For closed-circuit experiments, the FTR at the end of the perfusion period was considered.

Retention of dolutegravir in the placental cotyledon tissue was also determined, and expressed as an accumulation index (AI), defined by the ratio of the concentration of the study drug in the cotyledon (ng/mg) multiplied by the tissue density (1.04 g/mL) divided by the mean maternal drug concentration of the relevant experiment.

## Results

Five procedures were validated (see Supplementary data, S1 Table), with a mean ± standard deviation (SD) antipyrine FTR of 42% ± 17%.

**Fig 1:**
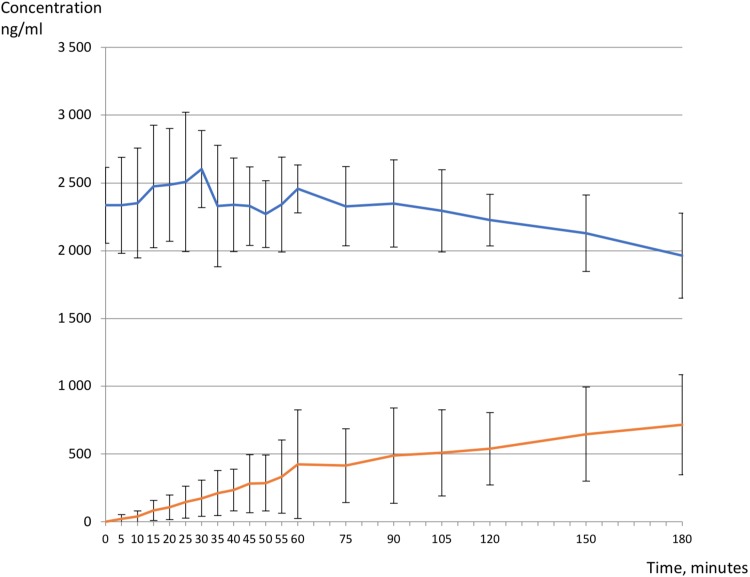
Dolutegravir concentration (ng/ml), mean (+/- SD) fetal (red line) and maternal (blue line) concentrations for all placentas in recirculating closed-circuit ex vivo human perfused cotyledon model, n = 5 experiments.

After 3h of perfusion (Fig 1), the mean (± SD) DTG concentrations in the maternal compartment and in the fetal compartment at 3h were respectively 2450 ± 286 ng/mL and 715 ± 369 ng/mL, with a mean fetal-to-maternal ratio of 34% ± 18% (Fig 1) and a clearance index of 79% ± 23%. The concentrations in the fetal compartment were well above the 90% inhibitory concentration (IC_90_) for wild-type HIV of 64 ng/ml for dolutegravir [29].

At the end of the recirculating/closed perfusion, the mean (SD) cotyledon accumulation index was 153% ± 25%.

## Discussion

Dolutegravir crossed the placenta, with a fetal-to-maternal ratio of 34% after 3 hours of perfusion in the closed-circuit model, at maternal concentrations in line with the in vivo average plasma concentrations obtained following once-daily oral administration 50 mg dolutegravir in HIV-infected patients. In that experiments, fetal concentrations exceeded the in vitro protein-binding adjusted IC90 values (64 ng/mL) [29]. We observed that dolutegravir accumulated in the placental lobule, suggesting that retention in the placenta, rather than efflux or metabolism of the substance by the placenta, may account for a large proportion of the free fraction, which is cleared from the maternal compartment.

The moderate molecular weight and lipophilic nature of dolutegravir (molecular weight of 419.129 g/mol, water solubility 95 mg/L at 25 °C and Octanol/Water Partition Coefficient of 2.2) [30], favor its placental transfer. On the other hand, dolutegravir is 99% bound to plasma proteins, mainly albumin, which is important because only the unbound form of the drug can equilibrate across the placental barrier [31]. Our group showed, for another highly protein-bound antiretroviral drug, the HIV protease inhibitor lopinavir, [32] that the FTR in the ex vivo placental perfusion model decreased as albumin concentrations in the perfusate were increased. Other groups have chosen not to add albumin at all to the perfusate, but to calculate theoretical in vivo concentrations accounting for high protein binding [15]. In the present study, we used a 2 g/liter concentration of human albumin in all experiments. Thus, the fetal exposure to dolutegravir is expected to be lower at physiological albumin concentrations.

When performing ex vivo placental perfusions without albumin, the transfer parameters for protein-binded molecules are modified. Schalkwijk et al [9] reported, using the same model but no albumin added, a higher transfer of dolutegravir in 6 placentas, with a fetal-to-maternal (mean+SD) concentration ratio of 60% ± 20% after 3 hours, with a maternal concentration in line with ours (2.3 ± 0.4 mg/L). Another possible reason for the higher transfer observed was a sharp increase at the end of the experiments, which might be related to alterations in the placental membrane after a prolonged perfusion period.

The placental transfer of dolutegravir seems to be higher as compared with the other integrase inhibitors, raltegravir and elvitegravir. For raltegravir, Vinot et al [33] found an FTR in the maternal-to-fetal direction of 9.1% ± 1.4% and a CLI of 0.28 ± 0.05%. For elvitegravir, we have reported in the same closed-circuit model, after 3h of perfusion an FTR of 20% ± 10% [20]. The placental transfer of dolutegravir appears to be higher than for HIV protease inhibitors [23] and lower than for nucleoside reverse transciptase inhibitors and HIV non-nucleoside reverse transcriptase inhibitors [27]. In contrast, there was no placental transfer of the fusion inhibitor enfuvirtide [14]. Since maternal-fetal transfer of dolutegravir, as well as other integrase inhibitors and protease inhibitors, involves the ATP-binding cassette (ABC) transporter p-glycoprotein (ABCB1), which is an efflux pump of drugs from the fetal to the maternal side [22], it was to be expected that placental transfer would be lesser than in case of simple diffusion.

The ex vivo cotyledon model is a non-invasive approach which has no ethical restrictions in order to study placental transfer of medications which have not yet been tested in pregnant women. It allows for evaluation in standardized and highly controlled conditions. Recirculating studies imitate physiological conditions and can be used to evaluate transplacental transfer and metabolism, while in open perfusions drug clearance can be calculated. Both of these approaches showed concordant results, and in addition the placental transfer appeared to be independent of the initial maternal concentration. Furthermore, placental accumulation, which is rarely analyzed in placental perfusion studies, is of interest to interpret placental metabolism and transport.

However, there are well-known limitations to the ex vivo perfusion model [13, 15, 34]. Only full-term placentas are used, which does not account for changes in placental morphology (thickness, surface area, and vascularization) and metabolic functions during gestation. Thus, the model does not explore fetal exposure in the post-conception period, which is the period at risk of teratogenesis. The ex vivo model does not fully take into account extra-placental factors such as protein binding discussed above and fetal metabolism and clearance.

## Conclusion

Placental transfer of dolutegravir is sufficient to entail in utero exposure of the fetus. Knowledge about placental transfer is important for clinical decision-making. High transfer to the fetus can protect it from perinatal transmission of HIV, acting as pre-exposure prophylaxis (PreP) [35], and dolutegravir’s long half-life could make it a candidate for intrapartum prophylaxis. However, in utero fetal may lead to drug-related toxicities and malformations [36, 37].

## Supporting information

S1 Table Results of validated recirculating placental perfusions of dolutegravir, n = 5. DTG: dolutegravir. ATP: antipyrine. CF: Concentration in fetal circulation. CM: Concentration in maternal circulation.
(XLSX)
